# Perspectives of Antidiabetic Drugs in Diabetes With Coronavirus Infections

**DOI:** 10.3389/fphar.2020.592439

**Published:** 2021-01-29

**Authors:** Bao Sun, Shiqiong Huang, Jiecan Zhou

**Affiliations:** ^1^ Department of Pharmacy, The Second Xiangya Hospital, Central South University, Changsha, China; ^2^ Institute of Clinical Pharmacy, Central South University, Changsha, China; ^3^ Department of Pharmacy, The First Hospital of Changsha, Changsha, China; ^4^ Institute of Clinical Medicine, The First Affiliated Hospital, University of South China, Hengyang, China

**Keywords:** diabetes mellitus, coronavirus infection, COVID-19, antidiabetic agents, therapeutic choices

## Abstract

Diabetes mellitus (DM) increases the risk of viral infections especially during the period of poor glycemic controls. Emerging evidence has reported that DM is one of the most common comorbidities in the novel severe acute respiratory syndrome coronavirus 2 (SARS-CoV2) infection, also referred to as COVID-19. Moreover, the management and therapy are complex for individuals with diabetes who are acutely unwell with suspected or confirmed COVID-19. Here, we review the role of antidiabetic agents, mainly including insulin, metformin, pioglitazone, dipeptidyl peptidase-4 (DPP4) inhibitors, sodium-glucose cotransporter 2 (SGLT2) inhibitors, and glucagon-like peptide 1 (GLP-1) receptor agonists in DM patients with coronavirus infection, addressing the clinical therapeutic choices for these subjects.

## Introduction

Coronavirus disease 2019 (COVID-19), caused by a novel severe acute respiratory syndrome coronavirus (SARS-CoV2), was declared to be a pandemic by the World Health Organization on March 11 and had aroused worldwide public concerns [https://www.who.int/dg/speeches/detail/who-director-general-s-opening-remarks-at-the-media-briefing-on-covid-19---11-march-2020 (2020)]. The global epidemic of SARS-CoV2 has direct implications for the therapy of common metabolic diseases such as diabetes mellitus (DM). Furthermore, DM is known to be associated with an increased risk of viral respiratory tract infections, including H1N1 influenza (Allard et al., 2010) and is emerging as an important comorbidity for disease severity and mortality in the context of COVID-19 (Targher et al., 2020; Yan et al., 2020). Strikingly, prevalence of DM was about twofold increase in the nonsurviving compared to the surviving COVID-19 individuals in China and Italy (Fadini et al., 2020a; Wu C. et al., 2020a), which was consistent with the independent association of this condition with fatal complications during the other two coronavirus-related respiratory infection epidemics, such as the Middle East Respiratory Syndrome (MERS) in 2012, and the Severe Acute Respiratory Syndrome (SARS) in 2002 (Zhou et al., 2020). Proposed mechanisms for these apparent associations between COVID-19 and DM may be attributed to the dysregulated immune response (Guo et al., 2020).

To date, the management of people with DM who are acutely unwell with COVID-19 is complex, and improved glycemic control should be of utmost importance in patients with COVID-19 and preexisting type 2 diabetes (Zhu et al., 2020). Although it would be wise to stick to the ongoing or intensive treatment, the choice of antidiabetic drugs needs to be reviewed. Herein, we summarize the role and perspective of antidiabetic agents, mainly including insulin, metformin, pioglitazone, dipeptidyl peptidase-4 (DPP4) inhibitors, sodium-glucose cotransporter 2 (SGLT2) inhibitors, and glucagon-like peptide 1 receptor agonists (GLP-1RAs) in DM patients with coronavirus infection.

## Associations Between DM and Coronavirus Infections

DM was correlated with an increased risk of viral respiratory tract infections (Allard et al., 2010) and was considered as a major contributor to disease severity and mortality in MERS (Memish et al., 2020). A systematic review and meta-analysis described that the overall prevalence of DM in MERS cases was 3.6-fold higher than in H1N1 (Badawi and Ryoo, 2016). Moreover, both smaller and larger studies revealed that DM was strongly associated with adverse outcomes and mortality in subjects with MERS (Assiri et al., 2013; Alqahtani et al., 2018). Similarly, a retrospective study performed by Booth et al. showed that the presence of DM was independently associated with significant morbidity and mortality in 114 adults hospitalized with SARS-CoV (Booth et al., 2003). Analysis of individuals hospitalized with SARS-CoV in China demonstrated that increases in fasting glucose were involved in the increased rates of death (Yang et al., 2010).

Database from Chinese Centers for Disease Control and Prevention (CDC) showed a diabetes prevalence of approximately 5% from the 20,982 patients with COVID-19 (Epidemiology Working Group for NCIP Epidemic Response, Chinese Center for Disease Control and Prevention, 2020). A report from Italy indicated nearly 17% diabetes prevalence from the 1043 COVID-19 patients (Grasselli et al., 2020). Noteworthily, available evidence from the CDC and hospitals indicated that the risk of fatal complications from COVID-19 was up to 50% higher in patients with DM than in those without (Remuzzi and Remuzzi, 2020). Moreover, the presence of typical complications of DM (heart failure and chronic kidney disease) increased COVID-19 mortality (Barron et al., 2020; Holman et al., 2020). Among 1,590 laboratory confirmed cases of COVID-19 from China, 8.2% of patients with DM yielded poorer clinical endpoints than those without (Guan et al., 2020). Consistent with these observations, DM is one of the comorbidities associated with adverse outcomes in hospitalized patients with SARS in China and Italy (Fadini et al., 2020a).

**FIGURE 1:**
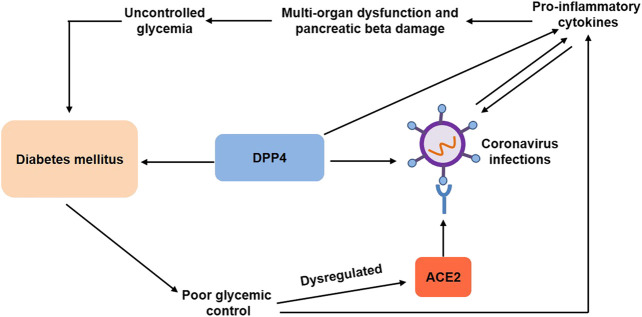
Associations between diabetes mellitus and coronavirus infections. Diabetes mellitus contributes to poor glycemic control, which has been shown to dysregulate ACE2 glycosylation and increase proinflammatory cytokines, facilitating viral cell entry. Preexisting proinflammatory state, in turn, predisposes patients to coronavirus infections and aggravates multiorgan dysregulation and pancreatic beta damage, leading to uncontrolled glycemia and diabetes mellitus. DPP4, a common pharmacological target for type 2 diabetes, is also a functional coronavirus receptor, which increases the susceptibility to coronavirus infections. On the other hand, DPP4 exerts proinflammatory activity. DPP4, dipeptidyl peptidase-4; ACE2, angiotensin converting enzyme 2.

Currently, there are mainly two specific mechanisms that might explain the link between DM and COVID-19 (Figure 1). First, both SARS and SARS-CoV2 coronavirus enter the body through angiotensin converting enzyme 2 (ACE2) and play a crucial role in metabolism and inflammation (Hoffmann et al., 2020). ACE2 has been identified as the receptor for the coronavirus. Poor glycemic control has been shown to dysregulate ACE2 glycosylation (Brufsky, 2020), which might facilitate viral cell entry or make the cells vulnerable to the inflammation (Hoffmann et al., 2020). Preexisting proinflammatory state accentuated the cytokine storm and was believed to contribute to multiorgan dysfunction and severity of diseases (Maddaloni and Buzzetti, 2020). In addition, the expression of ACE2 on pancreatic β cells could directly affect the β cell function (Yang et al., 2010; Roca-Ho et al., 2017), which might additionally worsen the clinical outcomes.

Second, dipeptidyl peptidase-4 (DPP4) enzyme, a common pharmacological target for type 2 diabetes, was also a functional coronavirus receptor (Raj et al., 2013), which might be another potential mechanism that explains the link between COVID-19 and DM. Transgenic mice expressing human DPP4 became susceptible to coronavirus infection with MERS-CoV (Li et al., 2016). Antibodies inhibited MERS-CoV infection of primary cells by directing against DPP4 (Raj et al., 2013). Analogously, recombinant human adenosine deaminase blocked MERS-CoV spike protein S1 binding to DPP4 and inhibited MERS-CoV infection of cells transfected with human DDP-4 (Raj et al., 2014). Moreover, human neutralizing antibodies directed against MERS-CoV spike protein blocked viral binding to DPP4, thereby inhibiting MERS-CoV infection (Tang et al., 2014). Surprisingly, transgenic mice overexpressing human DPP4 exhibited relative resistance to MERS-CoV infection and reduced rates of mortality (Algaissi et al., 2019). Although the association of SARS-CoV-2 and DDP-4 remains unknown, the use of DDP-4 inhibitors can provide therapeutic opportunities for the treatment of diabetic patients with COVID-19 in clinical practice (Iacobellis, 2020).

## Perspectives of Antidiabetic Agents in DM Patients With Coronavirus Infection

Considering the severity and mortality, extra precautions should be taken in DM patients with coronavirus infection. Of note, specific attention should be paid to the use of antidiabetic agents in these patients.

### Insulin

Insulin has been widely used for decades in critically ill hospitalized patients with DM and the usage of continuous glucose monitoring reduces the rates of hypoglycemia associated with insulin use (Lu et al., 2018). Of interest, insulin was also a preferred treatment option for critically ill patients with DM amid the COVID-19 pandemic (Drucker, 2020; Gupta et al., 2020). Additionally, selective loss of insulin action attenuated the anti-inflammatory T cell response to influenza infection in murine immune cells (Tsai et al., 2018). Furthermore, insulin played an important role in anti-inflammatory actions and reduced markers of inflammations in hospitalized patients with critical illness (Hansen et al., 2003). Intravenous insulin treatment had strong beneficial effects on inflammation and coagulation in hospitalized type 2 diabetic patients with COVID-19 over a period of 2 weeks (Sardu et al., 2020). As with other severe infection, diabetic ketoacidosis (DKA) has been reported in DM patients with COVID-19. Available evidence highlighted that subcutaneous insulin therapy was a useful strategy for uncomplicated DKA during the pandemic (Palermo et al., 2020). Particularly, Chen et al. showed that attention needed to be paid to patients with DM and COVID-19 who use insulin (Chen et al., 2020). They performed a retrospective study involving 904 patients with DM and COVID-19 and confirmed that insulin users had a greater risk of poor prognosis compared with noninsulin users (aOR 3.58 [95% CI 1.37, 9.35]; *p* = 0.009), but the study could not rule out the possible existence of truly uninfected patients among the clinically diagnosed cases (Chen et al., 2020).

Using the nonobese diabetic mice model, Heleia et al. reported that insulin downregulated ACE2 receptors (Roca-Ho et al., 2017), which might reduce the risk of viral infection. Moreover, an observational study revealed significantly higher insulin requirements among COVID-19 patients (Bornstein et al., 2020), which might be attributed to the beta-cell dysfunction induced by SARS-CoV2. Further research is required to clarify the clinical influence of insulin in the context of COVID-19.

### Metformin

Metformin, a first line antidiabetic drug in the treatment of type 2 diabetes, has anticipated antiproliferative and immunomodulatory effects. Previous studies suggested prohibiting metformin in patients with DM and COVID-19, due to an anticipated DKA in the context of multiorgan dysregulation (Puig-Domingo et al., 2020; Sinclair et al., 2020; Singh et al., 2020). Emerging evidence found that treatment with metformin in DM patients with coronavirus infection is not harmful and could possibly be beneficial (Kumar Singh and Singh, 2020). A multicenter study explored the association of blood glucose control and outcomes in patients receiving different antidiabetic agents with COVID-19 and found no harm with metformin (Zhu et al., 2020). In the Coronavirus Disease and Diabetes Outcome (CORONADO) trial, Bertrand et al. showed that only metformin users had a lower rate of death among all the antidiabetic agents, but the sample size and short-term prognosis (i.e., 7 days after admission) limited the credibility of the study (Cariou et al., 2020). Consistent with this result, Luo et al. performed a retrospective study including 283 patients with COVID-19 and suggested that in-hospital mortality was significantly lower in those receiving metformin compared with those not receiving (2.9% *vs*. 12.3%; *p* = 0.01) (Luo et al., 2020), but this finding might have been driven by selection bias, as patients with severe respiratory problems could not be treated with metformin. Noteworthily, metformin was recommended to be contraindicated in patients with or at risk of acidosis (Flory et al., 2020), and it should be discontinued if the glomerular filtration rate (GFR) was less than 30 ml per minute per 1.73 m^2^ [https://www.fda.gov/drugs/drug-safety-and-availability/fda-drug-safety-communication-fda-revises-warnings-regarding-use-diabetes-medicine-metformin-certain (2017)]. Recently, the guidelines for the management of diabetes during the COVID-19 pandemic addressed that it was recommended to stop treatment with metformin in those with fever and acute illness (body temperature >38.5 °C, GFR <30 ml/min/1.73 m^2^) (Sinclair et al., 2020).

Mechanistically, metformin activates AMP-activated protein kinase (AMPK) by causing its phosphorylation and regulates glucose and lipid metabolism (Zhou et al., 2001). Of note, as a downstream of AMPK, PI3K/AKT/mTOR pathway played major roles in MERS-CoV infection (Kindrachuk et al., 2015). Therefore, metformin may offer benefits in DM patients with coronavirus infection by indirectly mediating the mTOR pathway.

### Pioglitazone

Pioglitazone, a classical antidiabetic agent, has anti-inflammatory and antifibrotic activities (Radwan and Hasan, 2019). Studies have suggested that pioglitazone upregulated the expression of ACE2 (Zhang, et al., 2013), raising concerns about possible increased susceptibility to SARS-CoV2 infection (Pal and Bhadada, 2020). Furthermore, due to its adverse effects such as fluid retention (Alam et al., 2019), pioglitazone was recommended for discontinuation in acutely ill patients. In contrast, Mukherjee et al. considered that pioglitazone had more potential benefit than harm, and it could be continued in people with moderate COVID-19 (Jagat et al., 2020). Indeed, pioglitazone has been shown to decrease the secretion of various proinflammatory cytokines in the monocytes and macrophages (Bassaganya-Riera et al., 2010). Similarly, pioglitazone had the potential of blunting the cytokine storm by blocking caspase recruitment domain-containing protein 9 (CARD9) at the center of the immune activation mechanism in macrophages (Erol, 2020). Interestingly, computer-simulation-based bioinformatic analysis found that pioglitazone may target 3-chymotrypsin-like protease (3CLpro) and potentially inhibited SARS-CoV2 RNA synthesis and replication (Wu C. et al., 2020b). However, pioglitazone therapy was associated with weight gain and oedema and more importantly was associated with aggravation of heart failure (Kernan et al., 2016), which did not support the use of pioglitazone in patients with COVID-19. More clinical trials are needed to optimize the risk-benefit ratio of using pioglitazone in patients with COVID-19.

### DPP4 Inhibitors

DPP4, originally known as cluster of differentiation 26 (CD26), is a multifunctional soluble and cell-bound serine protease and plays critical roles in glucose homeostasis and inflammatory responses (Deacon, 2019). A previous study identified that DPP4 was a functional receptor for MERS-CoV (Raj et al., 2013) and may also participate in SARS-CoV2 infection despite not being its primary entry receptor. Targeting DPP4 has been thus considered as a pharmacologically reasonable strategy in the case of severe respiratory diseases related to coronaviruses and COVID-19 (Reinhold and Brocke, 2014; Iacobellis, 2020). It was also noteworthy that DPP4 was also involved in inflammatory and immune functions (Trzaskalski et al., 2020). Studies have proved that sitagliptin, one of the DPP4 inhibitors, was believed to reduce levels of proinflammatory markers such as tumor necrosis factor-α (TNF-α) and interleukin-6 (IL-6) (Matsubara et al., 2013; Satoh-Asahara et al., 2013). In this regard, DPP4 inhibitors might prevent coronaviruses infection and exert anti-inflammatory role. In a multicenter, retrospective study of the 338 consecutive patients with type 2 diabetes and COVID-19, sitagliptin treatment was associated with reduced mortality and improved clinical outcomes (Solerte et al., 2020). However, this retrospective study has several shortcomings, including the nonrandomized uncontrolled design, a slight increase in some of the inflammatory markers detected at baseline in the standard-of-care group as compared with the sitagliptin-treated patients, and the lack of some clinical data that were not available for all patients. Current knowledge did not all support the beneficial effects of DPP4 inhibitors on patients with diabetes and COVID-19. Recently, a retrospective study involving 904 patients with DM and moderate-severe COVID-19 showed that the use of DPP4 inhibitors did not significantly affect mortality and clinical outcomes (Chen et al., 2020). Another epidemiological study including 403 hospitalized COVID-19 patients found that DPP4 inhibitors might not affect the risk of hospitalization for COVID-19 patients with type 2 diabetes (Fadini et al., 2020b). A case series involving 387 COVID-19 patients in Italy described the association between DPP4 inhibitors treatment and a statistically reduced mortality, but the result was based on only 11 patients (Mirani et al., 2020). Of note, DPP4 inhibitors treatment was associated with worse outcomes in 27 patients with type 2 diabetes treated with DPP4 inhibitors than in 49 patients treated with other glucose-lowering drugs (Dalan et al., 2020). Consequently, there are some essential issues to be addressed before claiming possible beneficial effects of DPP4 inhibitors on COVID-19, and the effects of DPP4 inhibitors in patients with type 2 diabetes and COVID-19 should be confirmed in an ongoing randomized, placebo-controlled trial.

### SGLT2 Inhibitors

SGLT2 inhibitors were proposed as the second line treatment following metformin in the latest guidelines for the management of type 2 diabetes. Although several studies have discussed the potential benefits of SGLT2 inhibitors in COVID-19 patients (Chatterjee, 2020; Koufakis et al., 2020), the use of SGLT2 inhibitors was not beyond criticism. SGLT2 inhibitors were reported to increase ACE2 expression in kidney and therefore forming theoretical concern to increase susceptibility to SARS-CoV2 infection (Pal and Bhadada, 2020). Moreover, an expert panel recommended to avoid SGLT2 inhibitors among patients with DM and moderate-to-severe COVID-19 due to risk of dehydration and euglycemic DKA (Bornstein et al., 2020). Recently, Bossi et al. showed that SGLT2 inhibitors lacked efficacy in severe pneumonia related to novel coronavirus infection (Bossi et al., 2020). Conversely, SGLT2 inhibitors might exert anti-inflammatory effect in animal models (Bonnet and Scheen, 2018), which could favorably impact the dysregulated process in the context of cytokine storm of COVID-19. Intriguingly, dapagliflozin, a SGLT2 inhibitor, has been shown to decrease lactic acidosis and reverse acid-base balance inside the cells during hypoxia, thus contributing to prevent cell injury in the setting of cytokine storm of COVID-19 illness in patients with DM (Cure and Cure Cumhur, 2020). SGLT2 inhibitors have already been reported to provide a significant cardiorenal benefit, and thus they also might offer a protection to vital organs in the context of COVID-19. With these assumptions, “Dapagliflozin in Respiratory Failure in Patients with COVID-19” (DARE-19), a phase-3 multinational double-blind placebo-controlled randomized clinical trial (NCT04350593) has been initiated [https://www.clinicaltrials.gov/ct2/show/NCT04350593 (2020)]. Although SGLT2 inhibitors have been considered to provide benefits, they should be carefully reevaluated in case of body temperature >38.5°C or in case of food abstinence of insulin deficiency. Therefore, the potential benefit of SGLT2 inhibitors requires further validation.

### GLP-1RAs

GLP-1RAs, known as incretin mimetics, improve glucose homeostasis through enhancing glucose-dependent insulin secretion. Researchers found that liraglutide, the first long-acting GLP-1RAs, increased the expression of ACE2 in lungs and heart, which also raised a theoretical concern in patients with COVID-19 (Pal and Bhadada, 2020). Similar to DPP4 inhibitors, GLP-1RAs exerted anti-inflammatory effects by interfering with NF-kB signaling pathways (Lee and Jun, 2016). Furthermore, GLP-1RAs were associated with significant reduction in inflammatory cytokine in the respiratory epithelium in mice infected with respiratory syncytial virus (Bloodworth et al., 2018). Given that beneficial roles of GLP-1RAs for the prevention of cardiovascular and kidney diseases have been well established (Prattichizzo et al., 2019), these drugs could be an ideal option for the treatment of patients with DM at such risk (Ceriello et al., 2020). Of note, GLP-1RAs therapy was associated with reduction of hypoglycemia and glucose variability in the intensive care unit (ICU) setting, which could be protective in the critically ill patients (Mustafa and Whyte, 2019). However, initiating or maintaining such therapies in acute or critical situations (such as severe COVID-19) was not recommended because they will take time to become effective, due to slow uptitration, and might provoke nausea and vomiting (Nauck and Meier, 2019). There is insufficient evidence to clarify the use of GLP-1RAs in the context of the coronavirus infection. To date, no relative clinical-epidemiological studies have been carried out concerning the correlation between GLP-1RAs and COVID-19.

## Conclusion and Future Perspective

As available clinical evidence implicated diabetes as important risk factor impacting the severity of coronavirus infections, including SARS-CoV2, intensive monitoring and antidiabetic drug therapy should be considered in diabetic patients with COVID-19. We have attempted to highlight the potential benefits or risks of antidiabetic agents in the context of coronavirus infections (Table 1). Furthermore, we also addressed the clinical therapeutic choices of these agents for critically ill or moderate COVID-19 patients.

**TABLE 1:** Potential benefits or risks of antidiabetic agents in the context of coronavirus infections.

Antidiabetic agents	Beneficial or adverse effects	References	Recommendations
Insulin	Downregulated ACE2 receptors	Roca-Ho et al. (2017)	Preferred treatment options for critically ill patients
	Reduced inflammatory markers	Hansen et al. (2003), Sardu et al. (2020)	
	Reduced uncomplicated DKA	Palermo et al. (2020)	
	Increased the risk of poor prognosis	Chen et al. (2020)	
Metformin	Lowered deaths and interleukin-6 levels	Chen et al. (2020), Cariou et al. (2020)	Continued in mild to moderate COVID-19 and avoided in critically ill
	Lowered in-hospital mortality	Luo et al. (2020)	
	Targeted PI3K/AKT/mTOR pathways and inhibited viral replication	Kindrachuk et al. (2015)	
Pioglitazone	Upregulated ACE2 receptors	Zhang, et al. (2013)	Continued in mild to moderate COVID-19 and avoided in critically ill
	Decreased various proinflammatory cytokines	Bassaganya-Riera et al. (2010), Erol (2020)	
	Targeted 3CLpro and potentially inhibited SARS-CoV2 RNA synthesis and replication	Wu C. et al. (2020b)	
DPP4 inhibitors	Suppressed MERS-CoV infection	Reinhold and Brocke (2014)	Continued in mild to moderate COVID-19. More data needed for the acutely ill patients
	Reduced levels of proinflammatory markers	Satoh-Asahara et al. (2013), Matsubara et al. (2013)	
	Reduced mortality and improved clinical outcomes	Solerte et al. (2020)	
	Did not significantly affect mortality and clinical outcomes	Chen et al. (2020)	
	Might not affect the risk of hospitalization	Fadini et al. (2020a)	
	Associated with worse outcomes	Dalan et al. (2020)	
SGLT2 inhibitors	Upregulated ACE2 expression in kidney	Pal and Bhadada (2020)	Continued in mild to moderate COVID-19 and avoided in critically ill
	Exerted anti-inflammatory action and reduced cardiovascular and renal complications	Bonnet and Scheen (2018)	
	Decreased lactic acidosis and reversed acid-base balance inside the cells during hypoxia	Cure and Cure Cumhur (2020)	
GLP-1RAs	Increased the expression of ACE2 in lungs and heart	Pal and Bhadada (2020)	Continued in mild to moderate COVID-19. More data needed for the acutely ill patients
	Exerted anti-inflammatory effects and reduced inflammatory cytokine	Lee and Jun (2016), Bloodworth et al. (2018)	
	Reduced hypoglycemia and glucose variability	Mustafa and Whyte (2019)	

ACE2, angiotensin converting enzyme 2; DKA, diabetic ketoacidosis; COVID-19, coronavirus disease 2019; 3CLpro, 3-chymotrypsin-like protease; DPP4, dipeptidyl peptidase-4; SGLT2, sodium-glucose cotransporter 2; GLP-1RAs, glucagon-like peptide 1 receptor agonists.

Accumulative clinical studies have confirmed that DM was associated with a higher risk of severity and fatality of COVID-19 (Wu J. et al., 2020b; Zhang, et al., 2020), but few researchers clarified the influence of COVID-19 on DM. Remarkably, recent studies pointed that there was a bidirectional relationship between DM and COVID-19 (Rubino et al., 2020). New onset diabetes and severe metabolic complications of preexisting diabetes have been observed in patients with COVID-19 (Chee et al., 2020; Li et al., 2020), which posed challenges for clinical management of DM and suggested a complex pathophysiology of COVID-19-related diabetes. Thus, it is essential to investigate the epidemiologic features and pathogenesis of COVID-19-related diabetes and to gain clues regarding appropriate use of antidiabetic agents for patients during the COVID-19 pandemic.

Although current evidence has affirmed the role of antidiabetic agents in patients with COVID-19, it is not yet fully clear that these agents have a favorable or unfavorable effect. Nonetheless, well-controlled blood glucose is particularly crucial for DM patients with COVID-19 (Critchley et al., 2018; Wu J. et al., 2020a; Zhu et al., 2020). Therefore, it is essential to balance blood glucose control and avoid hyperglycemia or hypoglycemia during the use of antidiabetic agents. Noteworthily, a previous study indicated that insulin combined with continuous glucose monitoring (CGM) reduced hypoglycemia and proved to be safe and feasible (Breton et al., 2018). In this regard, antidiabetic agents combined with CGM might be a good treatment option for COVID-19 patients, particularly for the critical patients. On the other hand, another research showed that lixisenatide added to basal insulin significantly balanced blood glucose excursions without increasing the risk of hypoglycemia (Umpierrez et al., 2017). Thus, antidiabetic drugs combination might contribute to good blood glucose control and reduce adverse risks in moderate COVID-19 patients. Currently, there is only weak evidence to elucidate specific effects of antidiabetic drugs on COVID-19, and the retrospective analyses are subject to biases and unmeasured confounding. Further prospective randomized studies to confirm these therapeutic strategies are warranted.

Taken together, particular attention should be given to the safety concerns related to COVID-19 and the use of antidiabetic agents in patients with DM, and further clinical research in these domains will contribute to providing evidence-based therapies.

## Author Contributions

BS and JZ contributed to the supervision, concept, design, and revision of the article. All other authors contributed to the design and revision of the article. All authors gave final approval to submit the article for publication.

## Funding

This work was supported by the Fund Project of University of South China for Prevention and Control of COVID-19 (Nos. 2020-25 and 2020-26), Fund Project of Hunan Province for Prevention and Control of COVID-19 (Nos. 2020SK3010 and 2020SK3039), Fund Project of Hengyang City for Prevention and Control of COVID-19 (Nos. 2020hcjz6713 and 2020hcjz6716).

## Conflict of Interest

The authors declare that the research was conducted in the absence of any commercial or financial relationships that could be construed as a potential conflict of interest.

## References

[B1] Alam F. Islam M. A. Mohamed M. Ahmad I. Kamal M. A. Donnelly R. R. (2019). Efficacy and safety of pioglitazone monotherapy in type 2 diabetes mellitus: a systematic review and meta-analysis of randomised controlled trials. Sci. Rep. 9 (1), 5389 10.1038/s41598-019-41854-2 30926892 PMC6441028

[B2] Algaissi A. Agrawal A. S. Han S. Peng B. H. Luo C. Li F. F. (2019). Elevated human dipeptidyl peptidase 4 expression reduces the susceptibility of hDPP4 transgenic mice to Middle East respiratory syndrome coronavirus infection and disease. J. Infect. Dis. 219 (5), 829–835. 10.1093/infdis/jiy574 30256968 PMC6376904

[B3] Allard R. Leclerc P. Tremblay C. Tannenbaum T. N. (2010). Diabetes and the severity of pandemic influenza A (H1N1) infection. Diabetes Care 33 (7), 1491–1493. 10.2337/dc09-2215 20587722 PMC2890346

[B4] Alqahtani F. Y. Aleanizy F. S. Ali El Hadi Mohamed R. Alanazi M. S. Mohamed N. Alrasheed M. M. (2018). Prevalence of comorbidities in cases of Middle East respiratory syndrome coronavirus: a retrospective study. Epidemiol. Infect. 147, 1–5. 10.1017/s0950268818002923 PMC6518603 30394248

[B5] Assiri A. Al-Tawfiq J. A. Al-Rabeeah A. A. Al-Rabiah F. A. Al-Hajjar S. Al-Barrak A. (2013). Epidemiological, demographic, and clinical characteristics of 47 cases of Middle East respiratory syndrome coronavirus disease from Saudi Arabia: a descriptive study. Lancet Infect. Dis. 13 (9), 752–761. 10.1016/s1473-3099(13)70204-4 23891402 PMC7185445

[B6] Badawi A. Ryoo S. G. (2016). Prevalence of diabetes in the 2009 influenza A (H1N1) and the Middle East respiratory syndrome coronavirus: a systematic review and meta-analysis. J. Public Health Res. 5 (3), 733 10.4081/jphr.2016.733 28083520 PMC5206772

[B7] Barron E. Bakhai C. Kar P. Weaver A. Bradley D. Ismail H. (2020). Associations of type 1 and type 2 diabetes with COVID-19-related mortality in England: a whole-population study. The Lancet Diabetes & Endocrinology 8 (10), 813–822. 10.1016/s2213-8587(20)30272-2 32798472 PMC7426088

[B8] Bassaganya-Riera J. Song R. Roberts P. C. Hontecillas R. (2010). PPAR-gamma activation as an anti-inflammatory therapy for respiratory virus infections. Viral Immunol. 23 (4), 343–352. 10.1089/vim.2010.0016 20712478

[B9] Bloodworth M. H. Rusznak M. Pfister C. C. Zhang J. Bastarache L. Calvillo S. A. (2018). Glucagon-like peptide 1 receptor signaling attenuates respiratory syncytial virus-induced type 2 responses and immunopathology. J. Allergy Clin. Immunol. 142 (2), 683–e12. 10.1016/j.jaci.2018.01.053 29678751 PMC6078807

[B10] Bonnet F. Scheen A. J. (2018). Effects of SGLT2 inhibitors on systemic and tissue low-grade inflammation: the potential contribution to diabetes complications and cardiovascular disease. Diabetes Metab. 44 (6), 457–464. 10.1016/j.diabet.2018.09.005 30266577

[B11] Booth C. M. Matukas L. M. Tomlinson G. A. Rachlis A. R. Rose D. B. Dwosh H. A. (2003). Clinical features and short-term outcomes of 144 patients with SARS in the greater Toronto area. Jama 289 (21), 2801–2809. 10.1001/jama.289.21.JOC30885 12734147

[B12] Bornstein S. R. Rubino F. Khunti K. Mingrone G. Hopkins D. Birkenfeld A. L. (2020). Practical recommendations for the management of diabetes in patients with COVID-19. The Lancet Diabetes & Endocrinology 8 (6), 546–550. 10.1016/s2213-8587(20)30152-2 32334646 PMC7180013

[B13] Bossi A. C. Forloni F. Colombelli P. L. (2020). Lack of efficacy of SGLT2-i in severe pneumonia related to novel coronavirus (nCoV) infection: No little help from our friends. Diabetes Ther. 11 (7), 1605–1606. 10.1007/s13300-020-00844-8 32447736 PMC7244936

[B14] Breton M. D. Patek S. D. Lv D. Schertz E. Robic J. Pinnata J. (2018). Continuous glucose monitoring and insulin informed advisory system with automated titration and dosing of insulin reduces glucose variability in type 1 diabetes mellitus. Diabetes Technol. Therapeut. 20 (8), 531–540. 10.1089/dia.2018.0079 PMC6080127 29979618

[B15] Brufsky A. (2020). Hyperglycemia, hydroxychloroquine, and the COVID-19 pandemic. J. Med. Virol. 92 (7), 770–775. 10.1002/jmv.25887 32293710 PMC7262330

[B16] Cariou B. Hadjadj S. Wargny M. Pichelin M. Al-Salameh A. Allix I. (2020). Phenotypic characteristics and prognosis of inpatients with COVID-19 and diabetes: the CORONADO study. Diabetologia 63 (8), 1500–1515. 10.1007/s00125-020-05180-x 32472191 PMC7256180

[B17] Ceriello A. Stoian A. P. Rizzo M. (2020). COVID-19 and diabetes management: what should be considered? Diabetes Res. Clin. Pract. 163, 108151 10.1016/j.diabres.2020.108151 32305399 PMC7162752

[B18] Chatterjee S. (2020). SGLT-2 inhibitors for COVID-19– a miracle waiting to happen or just another beat around the bush? Prim Care Diabetes 14 (5), 564–565. 10.1016/j.pcd.2020.05.013 32493609 PMC7254022

[B19] Chee Y. J. Ng S. J. H. Yeoh E. (2020). Diabetic ketoacidosis precipitated by Covid-19 in a patient with newly diagnosed diabetes mellitus. Diabetes Res. Clin. Pract. 164, 108166 10.1016/j.diabres.2020.108166 32339533 PMC7194589

[B20] Chen Y. Yang D. Yang C. Zheng L. Huang K. Yang C. (2020). Response to Comment on Chen et al. Clinical Characteristics and Outcomes of Patients With Diabetes and COVID-19 in Association With Glucose-Lowering Medication. Diabetes Care 2020;43:1399-1407. Diabetes Care 43 (7), e165–1407. 10.2337/dc20-0660 32409498

[B21] Critchley J. A. Carey I. M. Harris T. DeWilde S. Hosking F. J. Cook D. G. (2018). Glycemic control and risk of infections among people with type 1 or type 2 diabetes in a large primary care cohort study. Diabetes Care 41 (10), 2127–2135. 10.2337/dc18-0287 30104296

[B22] Cure E. Cumhur Cure M. (2020). Can dapagliflozin have a protective effect against COVID-19 infection? A hypothesis. Diabetes & Metabolic Syndrome: Clin. Res. Rev. 14 (4), 405–406. 10.1016/j.dsx.2020.04.024 PMC7195078 32335366

[B23] Dalan R. Ang L. W. Tan W. Y. T. Fong S.-W. Tay W. C. Chan Y.-H. (2020). The association of hypertension and diabetes pharmacotherapy with COVID-19 severity and immune signatures: an observational study. European heart journal Cardiovascular pharmacotherapy 7, pvaa098 10.1093/ehjcvp/pvaa098 PMC7454507 32766831

[B24] Deacon C. F. (2019). Physiology and Pharmacology of DPP-4 in glucose homeostasis and the treatment of type 2 diabetes. Front. Endocrinol. 10, 80 10.3389/fendo.2019.00080 PMC6384237 30828317

[B25] Drucker D. J. (2020). Coronavirus infections and type 2 diabetes-shared pathways with therapeutic implications. Endocr. Rev. 41 (3), bnaa011 10.1210/endrev/bnaa011 32294179 PMC7184382

[B26] Epidemiology Working Group for NCIP Epidemic Response, Chinese Center for Disease Control and Prevention (2020). The epidemiological characteristics of an outbreak of 2019 novel coronavirus diseases (COVID-19) in China]. Zhonghua Liu Xing Bing Xue Za Zhi 41 (2), 145–151. 10.3760/cma.j.issn.0254-6450.2020.02.003 32064853

[B27] Erol A. (2020). Role of oxidized LDL-induced “trained macrophages” in the pathogenesis of COVID-19 and benefits of pioglitazone: a hypothesis. Diabetes & Metabolic Syndrome: Clin. Res. Rev. 14 (4), 713–714. 10.1016/j.dsx.2020.05.007 PMC7214326 32470851

[B28] Fadini G. P. Morieri M. L. Longato E. Avogaro A. (2020a). Prevalence and impact of diabetes among people infected with SARS-CoV-2. J. Endocrinol. Invest. 43 (6), 867–869. 10.1007/s40618-020-01236-2 32222956 PMC7103097

[B29] Fadini G. P. Morieri M. L. Longato E. Bonora B. M. Pinelli S. Selmin E. (2020b). Exposure to dipeptidyl‐peptidase‐4 inhibitors and COVID ‐19 among people with type 2 diabetes: a case‐control study. Diabetes Obes. Metabol. 22 (10), 1946–1950. 10.1111/dom.14097 PMC7283835 32463179

[B30] Flory J. H. Hennessy S. Bailey C. J. Inzucchi S. E. (2020). Reports of lactic acidosis attributed to metformin, 2015-2018. Diabetes Care 43 (1), 244–246. 10.2337/dc19-0923 31597667 PMC7011199

[B31] Grasselli G. Zangrillo A. Zanella A. Antonelli M. Cabrini L. Castelli A. (2020). Baseline characteristics and outcomes of 1591 patients infected with SARS-CoV-2 admitted to ICUs of the lombardy region, Italy. J. Am. Med. Assoc. 323 (16), 1574–1581. 10.1001/jama.2020.5394 PMC7136855 32250385

[B32] Guan W. J. Liang W. H. Zhao Y. Liang H. R. Chen Z. S. Li Y. M. (2020). Comorbidity and its impact on 1590 patients with COVID-19 in China: a nationwide analysis. Eur. Respir. J. 55 (5), 2000547 10.1183/13993003.00547-2020 32217650 PMC7098485

[B33] Guo W. Li M. Dong Y. Zhou H. Zhang Z. Tian C. (2020). Diabetes is a risk factor for the progression and prognosis of COVID‐19. Diabetes Metab. Res. Rev. 36, e3319 10.1002/dmrr.3319 PMC7228407 32233013

[B34] Gupta R. Ghosh A. Singh A. K. Misra A. (2020). Clinical considerations for patients with diabetes in times of COVID-19 epidemic. Diabetes & Metabolic Syndrome: Clin. Res. Rev. 14 (3), 211–212. 10.1016/j.dsx.2020.03.002 PMC7102582 32172175

[B35] Hansen T. K. Thiel S. Wouters P. J. Christiansen J. S. Van den Berghe G. (2003). Intensive insulin therapy exerts antiinflammatory effects in critically ill patients and counteracts the adverse effect of low mannose-binding lectin levels. J. Clin. Endocrinol. Metab. 88 (3), 1082–1088. 10.1210/jc.2002-021478 12629088

[B36] Hoffmann M. Kleine-Weber H. Schroeder S. Krüger N. Herrler T. Erichsen S. (2020). SARS-CoV-2 cell entry depends on ACE2 and TMPRSS2 and is blocked by a clinically proven protease inhibitor. Cell 181 (2), 271–e278. 10.1016/j.cell.2020.02.052 32142651 PMC7102627

[B37] Holman N. Knighton P. Kar P. O'Keefe J. Curley M. Weaver A. (2020). Risk factors for COVID-19-related mortality in people with type 1 and type 2 diabetes in England: a population-based cohort study. The Lancet Diabetes & Endocrinology 8 (10), 823–833. 10.1016/s2213-8587(20)30271-0 32798471 PMC7426091

[B38] Iacobellis G. (2020). COVID-19 and diabetes: can DPP4 inhibition play a role? Diabetes Res. Clin. Pract. 162, 108125 10.1016/j.diabres.2020.108125 32224164 PMC7271223

[B39] Jagat J. M. Kalyan K. G. Subir R. (2020). Use of pioglitazone in people with type 2 diabetes mellitus with coronavirus disease 2019 (COVID-19): boon or bane? Diabetes & metabolic syndrome 14 (5), 829–831. 10.1016/j.dsx.2020.06.015 32540737 PMC7836749

[B40] Kernan W. N. Viscoli C. M. Furie K. L. Young L. H. Inzucchi S. E. Gorman M. (2016). Pioglitazone after ischemic stroke or transient ischemic attack. N. Engl. J. Med. 374 (14), 1321–1331. 10.1056/NEJMoa1506930 26886418 PMC4887756

[B41] Kindrachuk J. Ork B. Hart B. J. Mazur S. Holbrook M. R. Frieman M. B. (2015). Antiviral potential of ERK/MAPK and PI3K/AKT/mTOR signaling modulation for Middle East respiratory syndrome coronavirus infection as identified by temporal kinome analysis. Antimicrob. Agents Chemother. 59 (2), 1088–1099. 10.1128/aac.03659-14 25487801 PMC4335870

[B42] Koufakis T. Zebekakis S. Ajjan P. Kotsa R. A. (2020). Sodium-glucose cotransporter 2 inhibitors in the era of COVID-19 pandemic: is the benefit to risk ratio still favorable? J. Diabetes Sci. Technol. 14 (4), 745–747. 10.1177/1932296820932155 32486846 PMC7673172

[B43] Kumar Singh A. Singh R. (2020). Is Metformin ahead in the race as a repurposed host-directed therapy for patients with diabetes and COVID-19? Diabetes Res. Clin. Pract. 165, 108268 10.1016/j.diabres.2020.108268 32533990 PMC7836896

[B44] Lee Y. S. Jun H. S. (2016). Anti-inflammatory effects of GLP-1-based therapies beyond glucose control. Mediat. Inflamm. 2016, 3094642 10.1155/2016/3094642 PMC4823510 27110066

[B45] Li J. Wang X. Chen J. Zuo X. Zhang H. Deng A. (2020). COVID ‐19 infection may cause ketosis and ketoacidosis. Diabetes Obes. Metabol. 22 (10), 1935–1941. 10.1111/dom.14057 PMC7264681 32314455

[B46] Li K. Wohlford-Lenane C. Perlman S. Zhao J. Jewell A. K. Reznikov L. R. (2016). Middle East respiratory syndrome coronavirus causes multiple organ damage and lethal disease in mice transgenic for human dipeptidyl peptidase 4. J. Infect. Dis. 213 (5), 712–722. 10.1093/infdis/jiv499 26486634 PMC4747621

[B47] Lu M. Zuo Y. Guo J. Wen X. Kang Y. (2018). Continuous glucose monitoring system can improve the quality of glucose control and glucose variability compared with point-of-care measurement in critically ill patients. Medicine 97 (36), e12138 10.1097/md.0000000000012138 30200106 PMC6133393

[B48] Luo P. Qiu L. Liu Y. Liu X. L. Zheng J. L. Xue H. Y. (2020). Metformin treatment was associated with decreased mortality in COVID-19 patients with diabetes in a retrospective analysis. Am. J. Trop. Med. Hyg. 103 (1), 69–72. 10.4269/ajtmh.20-0375 32446312 PMC7356425

[B49] Maddaloni E. Buzzetti R. (2020). Covid‐19 and diabetes mellitus: unveiling the interaction of two pandemics. Diabetes Metab. Res. Rev. 36, e33213321 10.1002/dmrr.3321 PMC7228318 32233018

[B50] Matsubara J. Sugiyama S. Akiyama E. Iwashita S. Kurokawa H. Ohba K. (2013). Dipeptidyl peptidase-4 inhibitor, sitagliptin, improves endothelial dysfunction in association with its anti-inflammatory effects in patients with coronary artery disease and uncontrolled diabetes. Circ. J. 77 (5), 1337–1344. 10.1253/circj.cj-12-1168 23386232

[B51] Memish Z. A. Perlman S. Van Kerkhove M. D. Zumla A. (2020). Middle East respiratory syndrome. Lancet 395 (10229), 1063–1077. 10.1016/s0140-6736(19)33221-0 32145185 PMC7155742

[B52] Mirani M. Favacchio G. Carrone F. Betella N. Biamonte E. Morenghi E. (2020). Impact of comorbidities and glycemia at admission and dipeptidyl peptidase 4 inhibitors in patients with type 2 diabetes with COVID-19: a case series from an academic hospital in lombardy, Italy. Diabetes Care 43 (12), 3042–3049. 10.2337/dc20-1340 33023989

[B53] Mustafa O. G. Whyte M. B. (2019). The use of GLP‐1 receptor agonists in hospitalised patients: an untapped potential. Diabetes Metab. Res. Rev. 35 (8), e3191 10.1002/dmrr.3191 31141838 PMC6899667

[B54] Nauck M. A. Meier J. J. (2019). Management OF endocrine disease: are all GLP-1 agonists equal in the treatment of type 2 diabetes? Eur. J. Endocrinol. 181 (6), R211–r234. 10.1530/eje-19-0566 31600725

[B55] Pal R. Bhadada S. K. (2020). Should antidiabetic medications be reconsidered amid COVID-19 pandemic? Diabetes Res. Clin. Pract. 163, 108146 10.1016/j.diabres.2020.108146 32283128 PMC7151403

[B56] Palermo N. E. Sadhu A. R. McDonnell M. E. (2020). Diabetic ketoacidosis in COVID-19: unique concerns and considerations. J. Clin. Endocrinol. Metab. 105 (8), dgaa360 10.1210/clinem/dgaa360 32556147 PMC7337869

[B57] Prattichizzo F. La Sala L. Rydén L. Marx N. Ferrini M. Valensi P. (2019). Glucose-lowering therapies in patients with type 2 diabetes and cardiovascular diseases. Eur. J. Prev. Cardiol. 26 (2), 73–80. 10.1177/2047487319880040 31766918

[B58] Puig-Domingo M. Marazuela M. Giustina A. (2020). COVID-19 and endocrine diseases. A statement from the European Society of Endocrinology. Endocrine 68 (1), 2–5. 10.1007/s12020-020-02294-5 32279224 PMC7150529

[B59] Radwan R. R. Hasan H. F. (2019). Pioglitazone ameliorates hepatic damage in irradiated rats via regulating anti-inflammatory and antifibrogenic signalling pathways. Free Radic. Res. 53 (7), 748–757. 10.1080/10715762.2019.1624742 31146611

[B60] Raj V. S. Mou H. Smits S. L. Dekkers D. H. Müller M. A. Dijkman R. (2013). Dipeptidyl peptidase 4 is a functional receptor for the emerging human coronavirus-EMC. Nature 495 (7440), 251–254. 10.1038/nature12005 23486063 PMC7095326

[B61] Raj V. S. Smits S. L. Provacia L. B. van den Brand J. M. Wiersma L. Ouwendijk W. J. (2014). Adenosine deaminase acts as a natural antagonist for dipeptidyl peptidase 4-mediated entry of the Middle East respiratory syndrome coronavirus. J. Virol. 88 (3), 1834–1838. 10.1128/jvi.02935-13 24257613 PMC3911594

[B62] Reinhold D. Brocke S. (2014). DPP4-directed therapeutic strategies for MERS-CoV. Lancet Infect. Dis. 14 (2), 100–101. 10.1016/s1473-3099(13)70696-0 PMC7128741 24457167

[B63] Remuzzi A. Remuzzi G. (2020). COVID-19 and Italy: what next? Lancet 395 (10231), 1225–1228. 10.1016/s0140-6736(20)30627-9 32178769 PMC7102589

[B64] Roca-Ho H. Riera M. Palau V. Pascual J. Soler M. J. (2017). Characterization of ACE and ACE2 expression within different organs of the NOD mouse. Int. J. Mol. Sci. 18 (3), 563 10.3390/ijms18030563 PMC5372579 28273875

[B65] Rubino F. Amiel S. A. Zimmet P. Alberti G. Bornstein S. Eckel R. H. (2020). New-onset diabetes in covid-19. N. Engl. J. Med. 383 (8), 789–790. 10.1056/NEJMc2018688 32530585 PMC7304415

[B66] Sardu C. D'Onofrio N. Balestrieri M. L. Barbieri M. Rizzo M. R. Messina V. (2020). Outcomes in patients with hyperglycemia affected by COVID-19: can we do more on glycemic control? Diabetes Care 43 (7), 1408–1415. 10.2337/dc20-0723 32430456 PMC7305003

[B67] Satoh-Asahara N. Sasaki Y. Wada H. Tochiya M. Iguchi A. Nakagawachi R. (2013). A dipeptidyl peptidase-4 inhibitor, sitagliptin, exerts anti-inflammatory effects in type 2 diabetic patients. Metab. Clin. Exp. 62 (3), 347–351. 10.1016/j.metabol.2012.09.004 23062489

[B68] Sinclair A. Dhatariya K. Burr O. Nagi D. Higgins K. Hopkins D. (2020). Guidelines for the management of diabetes in care homes during the Covid‐19 pandemic. Diabet. Med. 37 (7), 1090–1093. 10.1111/dme.14317 32369634 PMC7267536

[B69] Singh A. K. Gupta R. Ghosh A. Misra A. (2020). Diabetes in COVID-19: prevalence, pathophysiology, prognosis and practical considerations. Diabetes & Metabolic Syndrome: Clin. Res. Rev. 14 (4), 303–310. 10.1016/j.dsx.2020.04.004 PMC7195120 32298981

[B70] Solerte S. B. D'Addio F. Trevisan R. Lovati E. Rossi A. Pastore I. (2020). Sitagliptin treatment at the time of hospitalization was associated with reduced mortality in patients with type 2 diabetes and COVID-19: a multicenter, case-control, retrospective, observational study. Diabetes Care 43 (12), 2999–3006. 10.2337/dc20-1521 32994187 PMC7770266

[B71] Tang X. C. Agnihothram S. S. Jiao Y. Stanhope J. Graham R. L. Peterson E. C. (2014). Identification of human neutralizing antibodies against MERS-CoV and their role in virus adaptive evolution. Proc. Natl. Acad. Sci. U.S.A. 111 (19), E2018–E2026. 10.1073/pnas.1402074111 24778221 PMC4024880

[B72] Targher G. Mantovani A. Wang X.-B. Yan H.-D. Sun Q.-F. Pan K.-H. (2020). Patients with diabetes are at higher risk for severe illness from COVID-19. Diabetes Metab. 46 (4), 335–337. 10.1016/j.diabet.2020.05.001 32416321 PMC7255326

[B73] Trzaskalski N. A. Fadzeyeva E. Mulvihill E. E. (2020). Dipeptidyl peptidase-4 at the interface between inflammation and metabolism. Clin. Med. Insights Endocrinol. Diabetes 13, 1179551420912972 10.1177/1179551420912972 32231442 PMC7088130

[B74] Tsai S. Clemente-Casares X. Zhou A. C. Lei H. Ahn J. J. Chan Y. T. (2018). Insulin receptor-mediated stimulation boosts T cell immunity during inflammation and infection. Cell Metabolism 28 (6), 922–934. 10.1016/j.cmet.2018.08.003 30174303

[B75] Umpierrez G. E. O'Neal D. DiGenio A. Goldenberg R. Hernandez-Triana E. Lin J. (2017). Lixisenatide reduces glycaemic variability in insulin-treated patients with type 2 diabetes. Diabetes Obes. Metabol. 19 (9), 1317–1321. 10.1111/dom.12930 28256054

[B76] Wu C. Chen X. Cai Y. Xia J. Zhou X. Xu S. (2020a). Risk factors associated with acute respiratory distress syndrome and death in patients with coronavirus disease 2019 pneumonia in wuhan, China. JAMA Intern. Med. 180 (7), 934–943. 10.1001/jamainternmed.2020.0994 32167524 PMC7070509

[B77] Wu C. Liu Y. Yang Y. Zhang P. Zhong W. Wang Y. (2020b). Analysis of therapeutic targets for SARS-CoV-2 and discovery of potential drugs by computational methods. Acta Pharm. Sin. B. 10 (5), 766–788. 10.1016/j.apsb.2020.02.008 32292689 PMC7102550

[B78] Wu J. Huang J. Zhu G. Wang Q. Lv Q. Huang Y. (2020a). Elevation of blood glucose level predicts worse outcomes in hospitalized patients with COVID-19: a retrospective cohort study. BMJ Open Diab. Res. Care. 8 (1), e001476 10.1136/bmjdrc-2020-001476 PMC7298690 32503812

[B79] Wu J. Zhang J. Sun X. Wang L. Xu Y. Zhang Y. (2020b). Influence of diabetes mellitus on the severity and fatality of SARS‐CoV‐2 (COVID‐19) infection. Diabetes Obes. Metabol. 22 (10), 1907–1914. 10.1111/dom.14105 PMC7300679 32496012

[B80] Yan Y. Yang Y. Wang F. Ren H. Zhang S. Shi X. (2020). Clinical characteristics and outcomes of patients with severe covid-19 with diabetes. BMJ Open Diab. Res. Care. 8 (1), e001343 10.1136/bmjdrc-2020-001343 PMC7222577 32345579

[B81] Yang J. K. Lin S. S. Ji X. J. Guo L. M. (2010). Binding of SARS coronavirus to its receptor damages islets and causes acute diabetes. Acta Diabetol. 47 (3), 193–199. 10.1007/s00592-009-0109-4 19333547 PMC7088164

[B82] Zhang W. Li C. Liu B. Wu R. Zou N. Xu Y. Z. (2013). Pioglitazone upregulates hepatic angiotensin converting enzyme 2 expression in rats with steatohepatitis. Ann. Hepatol. 12 (6), 892–900. 10.1016/s1665-2681(19)31294-3 24114819

[B83] Zhang Y. Cui Y. Shen M. Zhang J. Liu B. Dai M. (2020). Association of diabetes mellitus with disease severity and prognosis in COVID-19: a retrospective cohort study. Diabetes Res. Clin. Pract. 165, 108227 10.1016/j.diabres.2020.108227 32446795 PMC7242190

[B84] Zhou F. Yu T. Du R. Fan G. Liu Y. Liu Z. (2020). Clinical course and risk factors for mortality of adult inpatients with COVID-19 in Wuhan, China: a retrospective cohort study. Lancet 395 (10229), 1054–1062. 10.1016/s0140-6736(20)30566-3 32171076 PMC7270627

[B85] Zhou G. Myers R. Li Y. Chen Y. Shen X. Fenyk-Melody J. (2001). Role of AMP-activated protein kinase in mechanism of metformin action. J. Clin. Invest. 108 (8), 1167–1174. 10.1172/jci13505 11602624 PMC209533

[B86] Zhu L. She Z. G. Cheng X. Qin J. J. Zhang X. J. Cai J. (2020). Association of blood glucose control and outcomes in patients with COVID-19 and pre-existing type 2 diabetes. Cell Metabol. 31 (6), 1068–e3. 10.1016/j.cmet.2020.04.021 PMC7252168 32369736

